# Comparison of the clinical characteristics and mortalities of severe COVID-19 patients between pre- and post-menopause women and age-matched men

**DOI:** 10.18632/aging.203532

**Published:** 2021-09-22

**Authors:** Danyong Liu, Han-Lin Ding, Yao Chen, De-Hong Chen, Changming Yang, Liu-Ming Yang, Jessica Aijia Liu, Liangqing Zhang, Zhong-Yuan Xia, Xi-He Zhang, Shaoqing Lei, Zhengyuan Xia

**Affiliations:** 1 Department of Anesthesiology, Affiliated Hospital of Guangdong Medical University, Zhanjiang, Guangdong, China; 2 State Key Laboratory of Pharmaceutical Biotechnology, The University of Hong Kong, Hong Kong, China; 3 The Department of Pain, Xiangyang Central Hospital, Affiliated Hospital of Hubei University of Arts and Science, Xiangyang, Hubei, China; 4 Department of Obstetrics, Affiliated Shenzhen Maternity and Child Healthcare Hospital, Southern Medical University, Shenzhen, Guangdong, China; 5 The Department of Urology, Xiangyang Central Hospital, Affiliated Hospital of Hubei University of Arts and Science, Xiangyang, Hubei, China; 6 Department of Anesthesiology, The First People’s of Hospital of Jingmen City, Jingmen, Hubei, China; 7 Guangdong Medical University Affiliated Lianjiang People’s Hospital, Lianjiang, Guangdong, China; 8 Department of Anesthesiology, The University of Hong Kong, Hong Kong, China; 9 Department of Anesthesiology, Renmin Hospital of Wuhan University, Wuhan, Hubei, China; 10 Shenzhen Institute of Research and Innovation, The University of Hong Kong, Shenzhen, Guangdong, China

**Keywords:** COVID-19, severely, mortality, menopausal, estrogen

## Abstract

The mortality rate of young female COVID-19 patients is reported to be lower than that of young males but no significant difference in mortality was found between female and male COVID-19 patients aged over 65 years, and the underlying mechanism is unknown. We retrospectively analyzed clinical characteristics and outcomes of severely ill pre- and post-menopausal COVID-19 patients and compared with age-matched males. Of the 459 patients included, 141 aged ≤55, among whom 19 died (16 males vs. 3 females, p<0.005). While for patients >55 years (n=318), 115 died (47 females vs. 68 males, p=0.149). In patients ≤55 years old, the levels of NLR, median LDH, median c-reactive protein and procalcitonin were significantly higher while the median lymphocyte count and LCR were lower in male than in female (all p<0.0001). In patients over 55, these biochemical parameters were far away from related normal/reference values in the vast majority of these patients in both genders which were in contrast to that seen in the young group. It is concluded that the mortality of severely ill pre-menopausal but not post-menopausal COVID-19 female patients is lower than age-matched male. Our findings support the notion that estrogen plays a beneficial role in combating COVID-19.

## INTRODUCTION

In December 2019, a series of unexplained cases of pneumonia were reported in Wuhan, Hubei, China, with clinical manifestations very similar to viral pneumonia [1]. Further investigation revealed that this disease was caused by the novel coronavirus SARS-CoV-2, and was subsequently officially named the 2019 coronavirus disease (COVID-19) by the World Health Organization (WHO) by 11. Feb 2020 [2]. The novel coronavirus SARS-CoV-2 has spread rapidly around the world, causing widespread concern. At present, the number of confirmed cases of COVID-19 in the world is still increasing rapidly. According to WHO, by the end of 2020, nearly 100 million patients worldwide have been diagnosed with COVID-19, with more than 2 million deaths.

Relevant research shows that the mortality rate of critically ill COVID-19 patients can be as high as 30-60% [3–5]. The incidence of serious diseases in female and young patients is lower than that of male and elderly patients [6, 7] and the overall mortality rate of female patients is also lower than that of male patients [3, 5]. Specifically, the mortality rate of young female COVID-19 patients is reported to be significantly lower than that of young male but no significant difference in mortality was found between female and male COVID-19 patients aged over 65 years old [7]. Preliminary reports from Europe [8] showed that women are more vulnerable to COVID- 19 infection in the 30-60-year age group relative to that in age matched men, while another study from Latin America [9] indicated that the overall case fatality rate of COVID- 19 is higher in men relative to that in women. However, neither of these studies specifically compared the case fatality of COVID-19 in pre- and post-menopause women relative to age matched men. However, there are relatively few researches that analyzed the relevant factors contributing to the gender differences in the outcome of severe COVID-19 patients about China, despite that estrogen has been presumably considered as a contributing factor.

17β-estradiol (E2), the main component of estrogen, has been used to treat COVID-19 [10]. However, the role of estrogen in affecting the mortality in severely ill COVID-19 patients is unclear. Given that both the incidence and mortality of severely ill COVID-19 are lower in young female than male, we thus presumed that the relatively higher levels of estrogen in young female patients conferred protective effects against COVID-19. The ovary is the main female reproductive organ that produces E2 and other essential female hormones. It begins to shrink and decline in function at the end of menopause. Previous studies have shown that the perimenopausal period of Chinese female is 44-55 years old, and the maximum age of menopause is about 55 years old [11, 12]. Thus, in this study, we retrospectively compared the clinical characteristics and outcomes of patients over 55 years old and those under or equal to 55 years old between the male and female patients with severe COVID-19, who were admitted to the Renmin Hospital of Wuhan University, Wuhan, Hubei, China and Xiangyang Central Hospital, Xiangyang, Hubei, China, as of April 1, 2020.

## MATERIALS AND METHODS

### Study design and participants

This study is a retrospective, cohort study conducted at Renmin Hospital of Wuhan University (Wuhan, China) and Xiangyang Central Hospital (Xiangyang, China), which were designated hospitals for the treatment of COVID-19 patients at the time of the pandemics. We conducted a retrospective analysis of severe patients who were diagnosed with COVID-19 according to the interim guidelines of the WHO [13] and who were enrolled from January 20, 2020 and discharged on or before April 1, 2020. The severity and clinical classification of COVID-19 were determined according to the Chinese COVID-19 Management Guidelines (Version 7.0). This study included critically ill patients on the basis of existing clinical classifications. Patients were diagnosed as severe cases when they met any of the following criteria: 1) shortness of breath, respiratory rate ≥ 30 breaths per minute; 2) pulse oxygen saturation ≤ 93% when in resting state; 3) the ratio of arterial partial pressure of oxygen to fraction of inspired oxygen (PaO2/FiO2) ≤ 300 mmHg; 4) pulmonary imaging showed that the lesions progressed significantly for more than 50% within 24-48 hours [14].

This study was approved by the Ethics Committee of Renmin Hospital of Wuhan University and Xiangyang Central Hospital. The informed consents were waived due to emerging infectious disease.

### Data collection

We collected data by reviewing electronic medical records, including basic feature data (gender, age), potential complications (hypertension, diabetes, cardiovascular disease, chronic lung disease, etc.), clinical symptoms and signs (fever, dry cough, dyspnea, etc.), laboratory test results (blood, biochemistry, cardiac biomarkers, coagulation function, etc.), treatment measures (antiviral, antibiotic, glucocorticoid therapy, continuous renal replacement therapy, oxygen support, extracorporeal membrane oxygenation, etc.) and Related complications (sepsis, arrhythmia, acute respiratory distress syndrome [ARDS], shock and acute kidney injury [AKI], etc.). The data were independently reviewed by two experienced researchers to verify the accuracy of the data.

### Outcomes

The main result was the difference in age, comorbidity, laboratory test results, and final outcomes between male and female patients in the different aged groups (i.e., aged >55 or ≤55) of severe patients. Secondary outcomes included: gender differences in the incidence of related complications between the two groups, such as the incidence of shock, ARDS, AKI, and arrhythmia. If the serum concentration of hs-TNI was in the 99th percentile upper reference limit, it was defined as acute heart injury. ARDS was diagnosed according to the Berlin Definition [15]. Sepsis was diagnosed according to the 2016 Third International Consensus Definition for Sepsis and Septic Shock [16]. AKI was identified on the basis of the Kidney Disease Improving Global Outcomes (KDIGO) criteria [17].

### Statistical analysis

After verification, it was found that the data belonged to skewed distribution, so the continuous variables were represented by the median (interquartile range, Q1-Q3), and the comparison was made by Mann-Whitney U test [18]. Categorical variables were expressed as (%) number, and were accurately tested and compared between the two groups of critically ill patients by chi-square or Fisher Differences for various data between male and female patients. And multiple regression analysis was used to compare the risk factors for death between the two groups. A two-sided p-value less than 0.05 was considered statistically significant. Data analyses were conducted with SPSS software (version 25.0) and GraphPad Prism (version 7.0).

## RESULTS

### Demographics and characteristics

As of April 1, 2020, The Renmin Hospital of Wuhan University and Xiangyang Central Hospital had admitted 1,752 patients with confirmed SARS-CoV-2 infection, of whom 1,293 patients did not meet the inclusion criteria. In this study, 459 severely ill patients were included. The median age of 459 patients was 64.0 years (52.0-73.0), ranging from 26 to 98 years, and 54.0% were male. A total of 135 severely ill patients (135/459, 29.4%) died during hospitalization, of whom 63.0% were male. All patients presented with bilateral involvement of typical chest computerized tomography (CT) manifestations of COVID-19 pneumonia upon admission. 247 patients (58.8%) had one or more comorbidities; the most common comorbidities were hypertension (174 [37.9%]), followed by diabetes (78 [17.0%]) and cardiovascular disease (72 [15.7%]). Fever (425 [92.6%]) is the most common symptom on admission, followed by dry cough (275 [59.9%]), dyspnea (270 [58.8%]), fatigue (194 [42.0%]), expectoration (154 [33.6%]), anorexia (94 [20%]), diarrhea (65 [14.2%]), abdominal pain (42 [9.2%]), sore throat (35 [7.6%]), dizziness (27 [5.9%]), headache (24 [5.2%]), nausea (23 [5.0%]), and vomiting (14 [3.1%]). Hemoptysis (6 [1.3%]) and abdominal pain (6 [1.3%]) were rare (Table 1). Of these patients (all=459), 141 patients aged ≤55 years old, with a median age of 47.0 years (38.0-52.0), and 54.6% were male; 318 patients aged older than 55 years, with a median age of 69.5 years (63.0-77.0), and 53.8% were male. (Table 1).

**Table 1:** Clinical characteristics among severe patients.

	**Total** **(N=459)**	**Severe patients ≤55 years old**				**Severe patients >55 years old**			
		**Sub-total** **(N = 141)**	**Male** **(n =77)**	**Female** **(n = 64)**	**P value ^a^**	**Sub-total** **(N = 318)**	**Male** **(n = 171)**	**Female** **(n = 147)**	**Pvalue ^b^**
Age, y	64 (52-73)	47 (38-52)	47 (41-52)	47 (38-71.75)	0.438	69.5 (63-77)	70 (63-78)	69 (64-76)	0.764
**Length of hospital stay, days**	15 (8-27)	18 (9-29)	17 (9-30)	18 (9-28)	0.566	14 (7-26)	15 (6-27)	13 (7-24)	0.521
survivors	22 (11-30)	20 (10-30)	23 (10.5-32)	19 (9-28)	0.146	24 (12.5-30)	25 (15.5-33.3)	20 (10.25-29)	0.013
non-survivors	6 (3-9)	7 (3-12)	7.5 (4.25-12.75)	3 (1-5)	0.138	6 (3-9)	6 (3-9)	7 (3-9)	0.765
**Comorbidities**	247 (53.8)	29 (20.6)	17 (22.1)	12 (18.8)	0.626	218 (68.6)	121 (70.8)	96 (65.3)	0.300
Hypertension	174 (37.9)	21 (14.9)	15 (19.5)	6 (9.4)	0.093	153 (48.1)	80 (46.8)	73 (49.7)	0.609
Diabetes	78 (17.0)	10 (7.1)	8 (10.4)	2 (3.1)	0.112	68 (21.4)	37 (21.6)	31 (21.1)	0.905
Cardiovascular disease	72 (15.7)	5 (3.5)	3 (3.9)	2 (3.1)	1.000	67 (21.1)	28 (16.4)	39 (26.5)	0.027
Chronic lung disease	29 (6.3)	2 (1.4)	2 (2.6)	0	0.501	27 (8.5)	16 (9.4)	11 (7.5)	0.550
Cerebrovascular disease	24 (5.2)	1 (0.7)	0	1 (1.6)	0.454	23 (7.2)	10 (5.8)	13 (8.8)	0.304
Chronic liver disease	21 (4.6)	5 (3.5)	4 (5.2)	1 (1.6)	0.377	16 (5.0)	10 (5.8)	6 (4.1)	0.473
Chronic kidney disease	19 (4.1)	4 (2.8)	3 (3.9)	1 (1.6)	0.626	15 (4.7)	9 (5.3)	6 (4.1)	0.620
Malignancy	13 (2.8)	NA	NA	NA	NA	13 (4.1)	8 (4.7)	5 (3.4)	0.566
**Signs and symptoms**									
Fever	425 (92.6)	129 (91.5)	71(92.2)	58 (90.6)	0.737	296 (93.1)	161 (94.2)	135 (91.8)	0.417
Dry cough	275 (59.9)	95 (67.4)	56 (72.7)	39 (60.9)	0.137	180 (56.6)	99 (57.9)	81 (55.1)	0.616
Dyspnea	270 (58.8)	66 (46.8)	44 (57.1)	22 (34.4)	0.007	204 (64.2)	122 (71.3)	102 (69.4)	0.794
Expectoration	154 (33.6)	46 (32.6)	26 (33.8)	20 (31.3)	0.751	108 (34)	69(40.4)	39 (26.5)	0.009
Fatigue	193 (42.0)	59 (41.8)	36 (46.8)	23 (35.9)	0.195	134 (42.1)	79 (46.2)	55 (37.4)	0.114
Anorexia	94 (20.5)	28 (19.9)	19 (24.7)	9 (14.1)	0.116	66 (20.8)	41 (24)	25 (17)	0.127
Diarrhea	65 (14.2)	24(17.0)	15 (19.5)	9 (14.1)	0.394	41 (12.9)	20 (11.7)	21 (14.3)	0.492
Myalgia	42 (9.2)	19 (13.5)	11 (14.3)	8 (12.5)	0.757	23 (7.2)	14 (8.2)	9 (6.1)	0.479
Pharyngalgia	35 (7.6)	16 (11.3)	9 (11.7)	7 (10.9)	0.889	19 (6.0)	11 (6.4)	8 (5.4)	0.710
Dizziness	27 (5.9)	4 (2.8)	4 (5.2)	0	0.064	23 (7.2)	8 (4.7)	15 (10.2)	0.058
Nausea	23 (5.0)	9 (6.4)	7 (9.1)	2 (3.1)	0.149	14 (4.4)	10 (5.8)	4 (4.4)	0.175
Headache	24 (5.2)	12 (8.5)	8 (10.4)	4 (6.3)	0.380	12 (3.8)	6 (3.5)	6 (4.1)	0.789
Vomiting	14 (3.1)	5 (3.5)	2 (2.6)	3 (4.7)	0.504	9 (2.8)	4 (2.3)	5 (3.4)	0.569
Hemoptysis	6 (1.3)	1 (0.7)	0	1 (1.6)	0.454	5 (1.6)	3 (1.8)	2 (1.4)	0.778
Abdominal pain	6 (1.3)	1 (0.7)	0	1 (1.6)	0.454	5 (1.6)	3 (1.8)	2 (1.4)	0.778
**Treatments**									
Antiviral therapy	410 (89.3)	122 (86.5)	66 (85.7)	56 (87.5)	0.757	288 (90.9)	157 (91.8)	131 (89.7)	0.521
Antibiotic therapy	365 (79.5)	108 (76.6)	59 (76.6)	49 (45.4)	0.993	257 (80.8)	143 (83.6)	114 (77.6)	0.170
Glucocorticoid therapy	168 (36.6)	53 (37.6)	35 (45.5)	18 (28.1)	0.034	115 (36.2)	70 (40.9)	45 (30.6)	0.056
Continuous renal replacement therapy	14 (3.1)	4 (2.8)	4 (5.2)	0	0.126	10 (3.2)	6 (3.5)	4 (2.7)	0.696
Oxygen inhalation	308 (67.1)	77 (54.6)	46 (59.7)	31 (48.4)	0.632	231 (72.6)	128 (74.9)	103 (70.1)	0.842
Noninvasive mechanical ventilation	51 (11.1)	15 (10.6)	13 (16.9)	2 (3.1)	0.008	36 (11.3)	23 (13.5)	13 (8.8)	0.196
Invasive mechanical ventilation	22 (4.8)	6 (4.3)	6 (7.8)	0	0.032	16 (5.0)	6 (3.5)	10 (6.8)	0.180
ECMO	2 (0.4)	1 (0.7)	1 (1.3)	0	1.000	1 (0.3)	0	1 (0.7)	0.280
**Complications**	225 (49.0)	38 (27.0)	29 (37.7)	9 (14.1)	0.002	187 (58.8)	103 (60.2)	84 (57.1)	0.577
ARDS	131 (28.5)	26 (18.4)	18 (23.4)	8 (12.5)	0.097	105 (33.0)	61 (35.7)	44 (29.9)	0.829
Sepsis	35 (7.6)	3 (2.1)	2 (2.6)	1 (1.6)	1.000	32 (10.1)	18 (10.5)	14 (9.5)	0.767
Acute kidney injury	18 (3.9)	3 (2.1)	3 (3.9)	0	0.251	15 (4.7)	6 (3.5)	9 (6.1)	0.273
Arrhythmia	14 (3.1)	2 (1.4)	2 (2.6)	0	0.501	12 (3.8)	6 (3.5)	6 (4.1)	0.789
Septic shock	27 (5.9)	4 (2.8)	4 (5.2)	0	0.126	23 (7.2)	12 (7.0)	11 (7.5)	0.873
**Prognosis**									
Discharge	324 (70.6)	122 (86.5)	61 (79.2)	61 (95.3)	..	203 (63.8)	103(60.2)	100 (68.0)	..
Death	135 (29.4)	19 (13.5)	16 (20.8)	3 (4.7)	0.005	115 (36.2)	68 (39.8)	47 (32.0)	0.149

Abbreviations: ARDS, acute respiratory distress syndrome; ECMO, extracorporeal membrane oxygenation;

^a^*P* values indicate differences between Male and Female ≤55 years old.

^b^*P* values indicate differences between Male and Female >55 years old.

*P* < 0.05 was considered statistically significant.

### Laboratory findings

The laboratory values on admission are summarized in Supplementary Table 1. The most common blood abnormalities in the entire cohort were lymphopenia (264/424 [62.3%]), and most patients had decreased T lymphocyte counts, including CD3 (214/319 [67.1%])`CD4 (204/319 [63.9%]) and CD8 (193/319 [60.5%]). This was followed by neutropenia (116/424 [27.4%]), mononucleosis (97/425 [22.8%]), and thrombocytopenia (35/423 [8.3%]). Most patients had elevated myocardial index, including lactate dehydrogenase (258/390 [66.2%]), N-terminal pro-B-type natriuretic (97/311 [31.2%]), and myoglobin (84/344 [24.4%]), hs-TNI (81/347 [23.3%]), creatine kinase-MB (CK-MB) (61/373 [16.4%]) and creatine kinase (45/392 [11.5%]). More than half of the patients had COVID-19-related inflammation, reflected by elevated c-reactive protein (CRP)(250/334 [74.9%]) and elevated procalcitonin (140/355 [39.5%]). Some patients had liver injury, indicated by elevated alanine aminotransferase (98/411 [23.8%]), aspartate aminotransferase (151/412 [36.7%]) and increased total bilirubin (30/414 [7.2%]). Increased blood urea nitrogen (107/414 [25.8%]) and increased serum creatinine (124/415 [29.9%]) indicated serious renal damage. Elevated blood glucose were seen in the majority of patients (180/268 [67.2%]). Coagulation dysfunction was also common: 267 of 394 patients (67.8%) had elevated d-dimer, 89 of 393 patients (22.6%) had prolonged activated partial thromboplastin time, 77 of the 396 cases (19.4%) had prolonged prothrombin time.

In patients aged ≤ 55 years old, the levels of lymphocyte and T lymphocyte counts in males were significantly lower, while the levels of alanine aminotransferase and aspartate aminotransferase, total bilirubin, urea nitrogen, creatinine, CRP, procalcitonin, LDH, Myoglobin, Creatine kinase were significantly higher than those in the female patients (Supplementary Table 1). For patients aged >55 years old the trends of changes in the levels of lymphocytes, T-lymphocyte counts and platelets as well as the levels of alanine aminotransferase, aspartate aminotransferase, total bilirubin, urea nitrogen, creatinine, CRP, procalcitonin, hs-TNI, Myoglobin, Creatine kinase and CK-MB and the differences between genders were basically the same as seen in patients aged ≤ 55 years old, while all these values in the aged patients deviated farther from the respective normal reference levels than that seen in patients aged ≤ 55 year old (Supplementary Table 1).

### Treatments, complications and clinical outcomes

**Figure 1:**
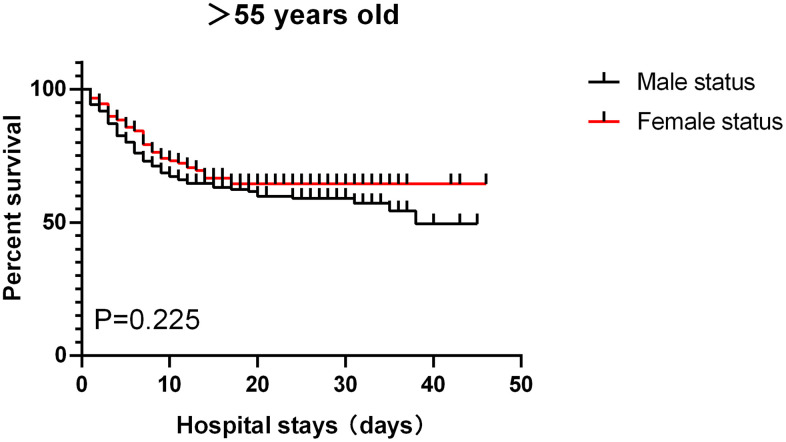
Patients aged >55, survival rate.

**Figure 2:**
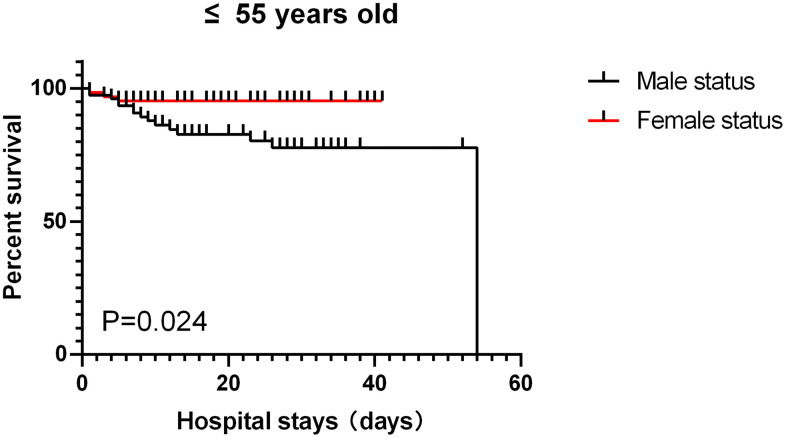
patients aged ≤ 55, survival rate.

Of the 459 patients, the vast majority (410 [89.3%]) of patients used antiviral drugs (eg, lopinavir, ritonavir), followed by antibiotics (365 [79.5%]) and glucocorticoids (168 [36.6%]). Only 6 patients (1.7%) received continuous renal replacement therapy (Table 1). 308 patients (67.1%) received oxygen inhalation, 51 patients (11.1%) received noninvasive mechanical ventilation,22 patients (4.8%) received invasive mechanical ventilation, and 2 patients received extracorporeal membrane oxygenation. Common complications included ARDS, coagulopathy, sepsis, acute kidney injury, arrhythmia, and septic shock (Table 1). Among patients aged ≤ 55 years old, male had relatively more complications than female but the differences did not reach statistical significance. For patients aged >55 years old, the incidence of cardiovascular diseases in female was significantly higher than that in male (p<0.05), while the incidences for all other complications such as diabetes and chronic lung disease did not significantly differ (Table 1). As shown in Table 1, the median hospital stay for the entire cohort was 15 days (8-27) and the length of hospital stay between groups did not significant differ. For the 141 patients aged ≤55, 122 were discharged (61 males and 61 females) and 19 died (16 males vs. 3 females, p=0.005). For the 318 patients aged >55, 203 were discharged (103 males vs. 100 females) and 115 died (68 males vs. 47 females, p=0.149). The overall survival rate was significant lower in male than in female in severely ill COVID-19 patients aged ≤55 (p<0.05) but not in patients aged >55 (p>0.1) (Figures 1, 2).

Univariate logistic regression analysis showed that gender, hypertension, chronic kidney disease, mechanical ventilation, ARDS, sepsis, septic shock were associated with nosocomial death in severe COVID-19 patients aged ≤55 years old. Hypertension, cardiovascular disease, cerebrovascular disease, glucocorticoid therapy, renal replacement therapy, mechanical ventilation, ARDS, sepsis, septic shock were associated with nosocomial death in severe COVID-19 patients aged > 55 years old. Multivariate logistic regression analysis results further showed that gender, hypertension and septic shock were associated with the incidence of nosocomial death in severe COVID-19 patients aged ≤55 years old. Cardiovascular disease, glucocorticoid therapy, ARDS, sepsis, and septic shock were associated with the incidence of nosocomial death in severe COVID-19 patients aged > 55 years old; (Supplementary Table 2).

## DISCUSSION

Studies have shown that the risk factors for adverse outcomes caused by COVID-19 include advanced age and the presence of comorbidities (such as hypertension, coronary heart disease, diabetes, COPD, nephrotic syndrome, etc.) [3, 4, 19]. We found that there is no significant difference in age and comorbidity between male and female patients in this cohort, but in the final outcome, the mortality rate in males is significantly higher than that of females in severe patients who aged ≤55 years old. The difference between the male and female patients in the laboratory test results is mainly manifested in the count of lymphocytes and T lymphocytes (CD3 / CD4 / CD8) and thrombocytopenia. Urea nitrogen, urea nitrogen, creatinine levels, D-dimer, CRP, procalcitonin, LDH, hs-TNI, Myoglobin, Creatine kinase, CK-MB were significantly increased in male and were significantly higher than those in female patients (both in patients aged ≤55 years old and >55 years old). Studies have shown that the above factors are related to the severity of the disease and high mortality [20], and our findings were consistent with the aforementioned findings. Our findings that the mortality rate in males is significantly higher than that of females in severe patients in whose who aged ≤55 years old but the mortality did not significantly differ between male and female aged >55 years old provide a strong clue that the female hormone estrogen should be a major beneficial factor in reducing mortality rate in severely ill COVID-19 patients.

It has been well known that COVID-19 not only causes pneumonia, but also damages other organs, such as the heart, liver and kidneys, as well as organ systems such as the blood and immune system [1, 21]. The patient eventually died of multiple organ dysfunction syndrome (MODS), shock, ARDS, heart failure, arrhythmia, and renal failure [19, 21, 22]. Studies have shown that COVID-19 susceptibility was associated with hyperfunction of inflammatory cells, which in response to COVID-19 infection causes cytokine storms, hypercoagulation, and lung and distal organ damage. COVID-19 vulnerability syndrome is an age-related disorder that is strictly biological age dependent and is associated with other age-related disorders [23]. Cytokine storms and immunosuppression are major causes of death in COVID-19 patients [24]. However, evidence regarding the role of female hormone in affecting the mortality rate in severely ill COVID-19 patients is lacking. This cohort found that female patients had lower disease severity and mortality than male patients especially in patients aged ≤55 years old. We believe that the influence of estrogen, especially E2, on the regulation of inflammatory response and immune cell function and cardiovascular system [25] may be one of the protective factors. According to reports, the incidence of cardiometabolic diseases in young female is usually much lower than that in male. However, middle-aged female lose this obvious protective effect during the menopausal transition period [26]. Estrogen has multiple effects due to the large tissue distribution of its receptors. The biological effect of estrogen is through its binding to two major estrogen receptors (ER): ERα and ERβ, leading to conformational changes, dimerization and co-activators entering the nucleus, and then they interact with estrogen response elements or other transcription factors Ligation, thereby regulating the transcription of the target gene [27]. ER signal transduction plays a key role in the innate and adaptive immune response during respiratory virus infection and tissue repair. In animal experiments, estrogen therapy can suppress inflammation and reduce virus titers, thereby improving survival rate [28]. Estrogen mainly exerts an anti-inflammatory effect by suppressing cytokine genes such as tumor necrosis factor-α (TNF-α) [29].

Among patients who aged ≤55 years old in this cohort, female patients had a much lower incidence of developing complications than male patients, especially in terms of lung injury (dyspnea, ARDS). However, this difference in incidence between males and females was not manifested among the patients aged more than 55 years old. The most possible explanation for this phenomenon is that the levels of estrogen in females after menopause were decreased, and thus the beneficial effects of estrogen seen in females younger than 55 no longer exist.

Estradiol-mediated lung inflammation control has been shown to be attributable to down-regulated NF-κB signaling. NF-κB is a transcription factor that directly regulates the expression of various pro-inflammatory cytokines (including TNF-α and IL-1β) [30]. It is known that high levels of estradiol can inhibit the transcription of pro-inflammatory genes through the NF-κB pathway [29]. Studies have shown that the anti-inflammatory properties of estradiol (at high systemic concentrations) can help prevent inflammation-related lung damage during H5N1 infection [31]. In addition, there are differences in the SARS-CoV-2 IgG antibody levels between male and female patients, which may be a potential reason for the difference in COVID-19 results between genders. In the early stages of the disease, the production of IgG antibodies in female patients in general has been shown to be stronger than in male patients [32].

The main target of SARS-CoV-2 invading the human body is the angiotensin converting enzyme 2 (ACE2) receptor. ACE2 is widely distributed in the human body, and is expressed in the heart, kidney, lung, liver, testis, and intestine [33, 34]. The gene encoding ACE2 is located on the X chromosome of Xp22.2 [35]. Therefore, in theory, women ’s susceptibility to SARS-CoV-2 virus increases. However, the severity of the disease and the incidence of adverse outcomes in young women are much lower than those of men, and less pregnant women who are infected with COVID-19 progressed to severely critical stages of the disease [36]. Studies have shown that estrogen has positive effects on cytokine release, neutrophil chemotaxis, HSP expression, HO-1 induction and organ function recovery after shock and sepsis, which may also be a factor contributing to the overall improved survival rate of female patients with severe COVID-19 [37]. In our current study, sepsis in general had significant impact on mortality in COVID-19 patients aged >55 years, but not in those ≤55, while septic shock had significant impact on mortality in COVID-19 patients aged ≤55. Findings of our research support the assumption that high levels of estrogen might dominate these protective effects. In addition, studies have shown that when the novel SARS-CoV-2 is combined with cellular ACE2, the ensuring proteolytic cleavage of the S protein by TMPRSS2 allows the fusion of the virus with the cell membrane [34, 38], while male Hormone-dependent modulation of lung TMPRSS2 expression may explain the increased susceptibility of men to develop severe SARS-CoV-2 infection [39], which is another factor for high mortality in male patients.

Human endocrine pancreas also express ACE2, so coronavirus may damage islets and cause acute hyperglycemia [40]. Therefore, in theory, SARS-Cov-2 infection may directly cause hyperglycemia. Hyperglycemia can directly or indirectly suppress immune function, resulting in absolute lymphopenia [41]. In addition, estrogen can improve insulin sensitivity and monocyte function, which may also be one of the protective factors. Among the patients ≤55 years old in this cohort, female patients had significantly lower blood glucose levels and abnormalities of monocytes than male; while in patients > 55 years old, this protective effect was not observed.

In short, estrogen, especially E2, provides a protective effect on the regulation of inflammatory response and immune cell function, the influence of the cardiovascular system, insulin sensitivity and the improvement of monocyte function, thus most female patients with COVID-19 was saved from death. However, this protective effect gradually weakens in female who have passed menopause. In addition, the WHO classified estrogens as carcinogenic in humans and one of the most important risk factors of breast cancer [42]. Breast cancer affects approximately 1 in 10 women and is the leading cause of death in females between the ages of 40 and 50 years in the Western world. Thus, the use of estrogen in the clinical treatment of severe COVID-19 patients may be more suitable for patients >55 years old but less or not suitable for female patients younger than 55. In addition, our findings showed that glucocorticoid was related to higher mortality of severe COVID-19 patients who aged >55 years old. It should be noted that currently available data from various clinical trial studies could not support definitive conclusions regarding whether or not patients with severe COVID-19 will benefit from the application of corticosteroids [43] and the differences in the treatment regimen and in primary end-points or duration of the observation may have made the comparisons between studies difficult, despite that overall administration of systemic corticosteroids seem to be associated with lower 28-day all-cause mortality [44]. Whether or not glucocorticoids when administrated in combination with estrogen might have a better protective effect in severely ill aged COVID-19 patients merits further studies.

This cohort study may have the following limitations. First, the study was conducted in only two hospitals with a limited sample size, which may affect our interpretation of the study results. Second, there may be selection bias in determining protective factors that affect clinical outcomes. Third, lack of or insufficient adherence to standard supportive care during difficult times may lead to poor clinical outcomes in some patients. A larger cohort study of patients with multiple centers or multiple classifications is also needed to validate our conclusions.

## Supplementary Material

Supplementary Table 1

Supplementary Table 2
