# Supplementary material for: Comparison of the clinical characteristics and mortalities of severe COVID-19 patients between pre- and post-menopause women and age-matched men
Source: Aging (Albany NY). 2021 Sep 22;13(18):21903–13. doi: 10.18632/aging.203532 (PMC8507288; doi:10.18632/aging.203532)
Supplement: Supplementary Table 1 [file aging-13-203532-s001.doc]

**Supplementary Table 1.** Initial laboratory findings in this cohort study.

| **Variables** | **Reference**  **range** | **Total**  **(N=459)** | **[S](../../../../C:/Users/13760/AppData/Local/youdao/dict/Application/8.9.2.0/resultui/html/index.html#/javascript:;)evere patients ≤55 years old** | | | | **[S](../../../../C:/Users/13760/AppData/Local/youdao/dict/Application/8.9.2.0/resultui/html/index.html#/javascript:;)evere patients ＞55 years old** | | | |
| --- | --- | --- | --- | --- | --- | --- | --- | --- | --- | --- |
|  |  |  | Sub-Total  (N = 141) | Male  (n =77) | Female  (n = 64) | P value ^a^ | Sub-Total  (N = 318) | Male  (n = 171) | Female  (n = 147) | P value ^b^ |
| **Hematologic** |  |  |  |  |  |  |  |  |  |  |
| White blood cell, ×10^9^/L | 3.5-9.5 | 5.745（4.40-8.00） | 5.4（4.16-7.78） | 5.72（4.39-8.07） | 5.1 (3.62-7.0) | 0.05 | 5.98（4.50-8.13） | 5.98（4.42-8.20） | 6.01 (4.57-7.94) | 0.922 |
| < 3.5 |  | 53/425（12.5） | 21/134（15.7） | 8/74 （10.8） | 13/59（22.0） | 0.086 | 32/291(11.0) | 18/163（11.0） | 14/128（10.9） | 0.977 |
| ≥ 3.5 to ≤9.5 |  | 306/425（72.0） | 96/134（71.6） | 54/74（73.0） | 42/59 (70.0) |  | 210/291(72.2) | 113/163（69.33） | 97/128（75.78） |  |
| > 9.5 |  | 66/425（15.5） | 17/134（12.7） | 12/74（16.2） | 5/59（8.5） |  | 49/291(16.8) | 32/163 (19.63) | 17/128 (13.28) |  |
| Neutrophil, ×10^9^/L | 1.8-6.3 | 4.17（2.79-6.53） | 3.37（2.43-6.11） | 3.77 (2.68-6.68) | 2.82 (1.97-4.56) | 0.008 | 4.45（3.08-6.82） | 4.62（3.13-7.32） | 4.44 (3.0-6.57) | 0.442 |
| > 6.3 |  | 116/424（27.4） | 30/133（22.6） | 21/74（28.4） | 9/59（15.3） | 0.072 | 86/291(29.6) | 51/163 (31.3) | 35/128 (27.3) | 0.464 |
| Lymphocyte, ×10^9^/L | 1.1-3.2 | 0.895（0.57-1.34） | 1.08（0.73-1.63） | 0.93(0.69-1.36) | 1.24 (0.84-1.83) | 0.004 | 0.83（0.52-1.27） | 0.75（0.48-1.04） | 1.02 (0.62-1.40) | ＜0.0001 |
| <0.8 |  | 178/424（42.0） | 42/133（31.6） | 31/74（41.9） | 11/59（18.6） | 0.004 | 136/291(46.7) | 91/163 (55.8) | 45/128 (35.2) | 0.0005 |
| NLR |  | 4.33(2.30-9.89) | 3.22 (1.74-5.56) | 3.84 (2.16-7.78) | 2.51 (1.47-3.91) | ＜0.0001 | 5.20 (2.76-12.55) | 6.36 (3.31-14.58) | 4.26 (2.24-9.98) | 0.003 |
| >3.13 |  | 276/413(66.8) | 69/132(52.3) | 45/73(61.6) | 24/59(40.7) | 0.02 | 207/281(73.7) | 127/153(83.0) | 80/128(62.5) | 0.004 |
| Monocyte, ×10^9^/L | 0.1-0.6 | 0.42（0.29-0.57） | 0.45（0.34-0.59） | 0.48 (0.36-0.61) | 0.41(0.32-0.53) | 0.037 | 0.40（0.28-0.57） | 0.40（0.28-0.59） | 0.42 (0.27-0.57) | 0.923 |
| ≤ 0.6 |  | 331/425（77.9） | 104/134（77.6） | 54/77（70.1） | 50/59（84.7） | 0.046 | 227/291(78.0) | 125/163(76.7) | 102/128（79.7） | 0.54 |
| > 0.6 |  | 97/425（22.8） | 33/134（24.6） | 23/77（29.9） | 9/59（15.3） |  | 64/291(22.0) | 38/163(23.3) | 26/128（20.3） |  |
| Platelet, ×10^9^/L | 100-400 | 200.5  （147-266） | 218  （161.25-293.75） | 213  (160.75-274.50) | 221  (160.50-318.25) | 0.326 | 196.5  （137-256.25） | 178  （121-241） | 204  (151-273) | 0.01 |
| < 100 |  | 35/423（8.3） | 4/133（1.7） | 3/74（4.1） | 1/58（1.7） | 0.436 | 31/290 (10.7) | 23/163 (14.1) | 8/127 (6.3) | 0.033 |
| CD3, /μL | 723-2737 | 509（297-841） | 568（424.5-994） | 517 (419-868) | 805 (437-1170) | 0.067 | 463.5（266-777.5） | 432（241.25-638.5） | 544.5 (306.5-851.5) | 0.017 |
| <723 |  | 214/319（67.1） | 59/105（56.2） | 39/59（66.1） | 20/46（43.5） | 0.020 | 155/214 (72.5) | 97/124(78.2) | 58/90 (64.4) | 0.026 |
| CD4, /μL | 404-1612 | 293（183-535） | 374（226.5-613） | 308 (204-510) | 423 (239.75-744.50) | 0.057 | 278（145.75-503） | 248.5（134.75-383.75） | 325. 5 (186.5-582.25) | 0.010 |
| <404 |  | 204/319（63.9） | 60/105（57.1） | 37/59（62.7） | 23/46 (50.0) | 0.192 | 144/214 (67.3) | 94/124 (75.7) | 50/90 (55.6) | 0.002 |
| CD8, /μL | 220-1129 | 175（94-296） | 244（139-367） | 205 (126-314) | 324 (173.75-426) | 0.011 | 162（71.25-260.5） | 143.5（62.5-232.25） | 172.5 (91.75-283.75) | 0.042 |
| <220 |  | 193/319（60.5） | 48/105（45.7） | 32/59 (54.2) | 16/46 (34.8) | 0.047 | 145/214（67.8） | 90/124（72.6） | 55/90 (61.1) | 0.076 |
| **Biochemical** |  |  |  |  |  |  |  |  |  |  |
| ALT, U/L | 7-40 | 25（17-39） | 23.5（15.25-46） | 33 (20.75-60.75) | 18 (11-31.25) | ＜0.0001 | 25（18-37） | 28（19-46） | 23 (15.75-29) | ＜0.0001 |
| > 40 |  | 98/411（23.8） | 34/128（26.6） | 24/70 (34.3) | 10/58 (17.2) | 0.030 | 64/283(22.6) | 50/157(31.8) | 14/126 (11.1) | ＜0.0001 |
| AST, U/L | 13-35 | 30（20.25-42.75） | 28（17-39） | 31(23-47) | 19.5(15-31.25) | ＜0.0001 | 30（22-43） | 35（23.5-52） | 25.5 (21-37) | ＜0.0001 |
| > 35 |  | 151/412（36.7） | 41/129（31.8） | 30/71(42.3） | 11/58（19.0） | 0.005 | 110/283(38.9) | 74/157(47.1) | 36/126 (28.6) | 0.001 |
| Total bilirubin, μmol/L | ≤ 23 | 10.45（7.38-15.23） | 9.3（6.9-12.6） | 11(8.2-15) | 7.45(5.25-9.65) | ＜0.0001 | 11.3（7.55-16.3） | 12.6（7.83-19.08） | 10.1 (7.5-15.1) | 0.015 |
| > 23 |  | 30/414（7.2） | 3/129（2.3） | 2/71（2.8） | 1/58（1.7） | 1.000 | 27/285(9.5) | 19/158(12) | 8/127 (6.3) | 0.101 |
| Blood urea nitrogen, mmol/L | 3.6-9.5 | 5.11（3.90-7.69） | 4.2(3.2-5.2） | 4.8(3.95-5.78) | 3.27 (2.60-4.40) | ＜0.0001 | 5.86（4.29-8.75） | 7.07（4.93-10.32） | 5.06 (3.8-6.96) | ＜0.0001 |
| > 9.5 |  | 69/414（16.7） | 7/131（5.3） | 5/72（12.5） | 2/59（3.4） | 0.061 | 62/283 (21.9) | 46/157 (44.6) | 16/126 (20.6) | 0.0008 |
| Creatinine, μmol/L | 41-73 | 63（52-77） | 61(50-73.5) | 72 (61-77) | 48.4 (40-58) | ＜0.0001 | 65（53-80.75） | 71（60-91.38） | 54.6 (47-67.03) | ＜0.0001 |
| >73 |  | 124/415（29.9） | 33/131（25.2） | 30/72（41.67） | 3/59（5.1） | ＜0.0001 | 91/284 (32) | 69/158 (43.7) | 22/126 (17.5) | ＜0.0001 |
| Glucose, mmol/L | 3.9-6.1 | 7.35（5.80-10.38） | 6.82(5.50-9.0) | 6.9(5.65-9.89) | 6.0 (5.2-8.28) | 0.265 | 7.4（5.9-10.5） | 7.5（5.8-10.5） | 7.4 (5.9-10.73) | 0.845 |
| > 6.1 |  | 180/268（67.2） | 44/75（58.7） | 30/45（66.7） | 14/30（46.7） | 0.085 | 136/193 (70.5) | 71/103 (68.9) | 65/90 (72.2) | 0.617 |
| **Heart-related biomarkers** |  |  |  |  |  |  |  |  |  |  |
| LDH, U/L | 120-250 | 291（222-421） | 263(194.25-335.25) | 291(249-423) | 204 (182-264) | ＜0.0001 | 320.5（233.25-451.75） | 327（241.5-458.5） | 297 (227-439) | 0.274 |
| > 250 |  | 258/390（66.2） | 70/122（57.4） | 50/67（74.6） | 20/55（36.4） | ＜0.0001 | 188/268 (70.1) | 108/149(72.5) | 80/119 (67.2) | 0.35 |
| Myoglobin, μg/L | ≤ 110 | 54.16（32.3-108.5） | 32.82(23.33-66.05) | 46.79(32.0-88.4) | 25.04 (16.99-29.65) | ＜0.0001 | 62.59（36.47-125.55） | 79.25(48.935-178.16) | 42.29 (29.40-86.9) | ＜0.0001 |
| > 110 |  | 84/344（24.4） | 11/95（18.6） | 8/53(15.1) | 3/41 (7.32) | 0.258 | 73/249(29.3) | 52/140(37.1) | 21/109 (19.3) | 0.002 |
| Creatine kinase, U/L | 30-180 | 65 (39-126) | 53(33-104) | 84.5(46.3-247.3) | 38 (31-53) | ＜0.0001 | 68（43-133.5） | 85(50-201.5) | 59 (37-94) | ＜0.0001 |
| ≥ 180 |  | 74/392（18.9） | 22/123（17.9） | 20/68（29.4） | 2/55（3.6） | 0.0002 | 52/269（19.3） | 41/150(26.7) | 11/119 (3.4) | 0.001 |
| CK-MB, ng/mL | ≤5 | 1.31(0.77-2.965) | 0.74(0.48-1.57) | 1(0.53-2.31) | 0.64 (0.425-1.12) | 0.049 | 1.57（0.94-3.42） | 1.855(0.97-4.30) | 1.50 (0.88-2.66) | 0.045 |
| > 5 |  | 61/373（16.4） | 14/107（13.1） | 10/62（16.1） | 4/45（8.9） | 0.273 | 47/266 (17.7) | 32/148(21.6) | 15/118 (12.7) | 0.058 |
| hs-TNI, pg/mL | ≤0.04 | 0.006  (0.006-0.034) | 0.006  (0.006-0.006) | 0.006  （0.006-0.006） | 0.006  （0.006-0.006） | 0.265 | 0.011 （0.006-0.060） | 0.016 (0.006-0.102) | 0.007  (0.006-0.032) | 0.013 |
| > 0.04 |  | 81/347（23.3） | 6/95（6.3） | 4/54（7.3） | 2/41（4.9） | 1.000 | 75/252 (29.8) | 52/141 (36.9) | 23/111 (20.7) | 0.005 |
| NT-ProBNP, pg/mL | ≤450 | 175.7(58.71-566.5） | 49.69 (19.35-148.35) | 51.58 (16.2-157.7) | 47.8 (22.1-133.5) | 0.539 | 317.7 (103.2-799.7) | 319.5 (88.17-817) | 293.65 (116.78-743.85) | 0.698 |
| > 450 |  | 97/311（31.2） | 6/88（6.8） | 3/47（6.4） | 3/41（7.32） | 1.000 | 91/223（40.8） | 49/123 (39.8) | 42/100 (42.0) | 0.744 |
| **Infection-related indices** |  |  |  |  |  |  |  |  |  |  |
| C-reactive protein, mg/L | ≤5 | 44.5  （9.85-89.4） | 23.8  (5.5-60.9) | 33.4  (13.8-82.5) | 6.6  （5.0-40.5） | ＜0.0001 | 56.5  （12.75-98.45） | 61.5  (18.4-110) | 44.35  (6.45-90.8) | 0.041 |
| > 5 |  | 268/334（80.2） | 71/93（76.3） | 53/59（91.4） | 18/34（52.9） | ＜0.0001 | 197/241（81.7） | 118/139（84.9） | 79/102（77.5） | 0.14 |
| LCR |  | 0.017(0.008-0.099) | 0.037(0.014-0.214) | 0.023(0.009-0.087) | 0.149(0.030-0.393) | ＜0.0001 | 0.015(0.006-0.082) | 0.011(0.005-0.050) | 0.019(0.009-0.130) | ＜0.001 |
| Procalcitonin, ng/mL | 0.02-0.05 | 0.072  （0.040-0.175） | 0.050  (0.034-0.109) | 0.074  (0.047-0.132) | 0.038  (0.021-0.049) | ＜0.0001 | 0.083  (0.043-0.212) | 0.109  (0.050-0.343) | 0.059  (0.032-0.153) | ＜0.0001 |
| ≤ 0.02 |  | 19/355（5.4） | 11/105（10.5） | 0/59（0） | 11/46（23.9） | ＜0.0001 | 8/250（3.2） | 1/140 (0.7) | 7/110 (6.4) | 0.013 |
| > 0.02 to < 0.05 |  | 109/355（30.7） | 41/105（39.0） | 17/59（28.8） | 24/46（52.2） |  | 68/250（27.2） | 33/140 (23.6) | 35/110 (31.8) |  |
| ≥ 0.05 |  | 227/355（63.9） | 53/105（50.5） | 42/59（71.2） | 11/46（23.9） |  | 174/250（69.6） | 106/140 (75.7) | 68/110 (61.8) |  |
| **Coagulation function** |  |  |  |  |  |  |  |  |  |  |
| PT, s | 9-13 | 12.1（11.5-12.8） | 12.1 (11.25-12.7) | 12.15 (11.4-12.7) | 11.8 (11.2-12.6) | 0.299 | 12.2 (11.6-13) | 12.4 (11.8-13.25) | 11.95 (11.5-12.7) | ＜0.0001 |
| ≤ 13 |  | 319/396（80.6） | 105/121（86.8） | 60/70（85.7） | 45/51（88.2） | 0.686 | 214/275 (77.8) | 109/153 (71.2) | 105/122 (86.1) | 0.003 |
| > 13 |  | 77/396（19.4） | 16/121（13.2） | 10/70（14.3） | 6/51（11.8） |  | 61/275 (22.2) | 44/153 (28.8) | 17/122 (13.9) |  |
| APTT, s | 21-35 | 28.1（25.9-31.05） | 27.9 (25.9-30.38) | 28.7 (26.15-30.95) | 27.2 (25.1-29.0) | 0.019 | 28.3（25.9-31.4） | 28.8 (26.7-32.5) | 27.35 (24.8-30.93) | ＜0.0001 |
| < 35 |  | 371/393（94.4） | 115/120（95.8） | 65/69（94.2） | 50/51（98.0） | 0.393 | 256/273 (93.8) | 140/151 (92.7) | 116/122 (95.1) | 0.421 |
| ≥ 35 |  | 22/393（5.6） | 5/120（4.2） | 4/69（5.8） | 1/51（2.0） |  | 17/273 (6.2) | 11/151 (7.3) | 6/122 (4.9) |  |
| D-dimer, mg/L | ≤ 0.55 | 0.85（0.46-3.80） | 0.51（0.25-0.95） | 0.53（0.33-1.01） | 0.44（0.23-0.95） | 0.112 | 1.19（0.58-4.66） | 1.09（0.56-4.55） | 1.25（0.61-5.23） | 0.882 |
| ≤ 0.55 |  | 127/394（43.2） | 65/120（54.2） | 35/69（50.7） | 30/51（58.8） | 0.422 | 62/274（22.6） | 36/152 (23.68) | 26/122（21.3） | 0.64 |
| 0.55< to ≤1.0 |  | 92/394（23.4） | 27/120（22.5） | 17/69（24.6） | 10/51（19.6） |  | 65/274（23.7） | 37/152 (24.3) | 28/122（23.0） |  |
| > 1.0 |  | 175/394（44.4） | 28/120（23.3） | 17/69（24.6） | 11/51（21.6） |  | 147/274（53.6） | 79/152 (52.0) | 68/122（55.7） |  |

Abbreviations: NLR:The value of neutrophil-tolymphocyte ratio; AST, aspartate aminotransferase; ALT, Alanine aminotransferase; CK-MB, creatine kinase myocardial isoenzyme; hs-TNI, high-sensitivity cardiac troponin I; LDH, Lactate dehydrogenase; NT-proBNP, N-terminal pro-B-type natriuretic peptide; LCR:The value of lymphocyte-to C-reactive protein ratio; PT, prothrombin time;APTT,Activated partial thromboplastin time.

^a^*P* values indicate differences between Male and Female ≤55 years old.

^b^*P* values indicate differences between Male and Female >55 years old.

*P* < 0.05 was considered statistically significant.
