# Supplementary material for: Comparison of the clinical characteristics and mortalities of severe COVID-19 patients between pre- and post-menopause women and age-matched men
Source: Aging (Albany NY). 2021 Sep 22;13(18):21903–13. doi: 10.18632/aging.203532 (PMC8507288; doi:10.18632/aging.203532)
Supplement: Supplementary Table 2 [file aging-13-203532-s002.doc]

**Supplementary Table 2. Logistic regression analysis of mortality related risk factors in severe  patients.**

| [V](../../../../C:/Users/13760/AppData/Local/youdao/dict/Application/8.9.6.0/resultui/html/index.html#/javascript:;)ariate | **Severe patients ≤55 years old** | | | | **Severe patients ＞55 years old** | | | |
| --- | --- | --- | --- | --- | --- | --- | --- | --- |
|  | Univariate  OR (95% CI) | P value ^a^ | Multivariate  OR (95% CI) | P value ^b^ | Univariate  OR (95% CI) | P value ^c^ | Multivariate  OR (95% CI) | P value ^d^ |
| [G](../../../../C:/Users/13760/AppData/Local/youdao/dict/Application/8.9.6.0/resultui/html/index.html#/javascript:;)ender (Fe[male](../../../../C:/Users/13760/AppData/Local/youdao/dict/Application/8.9.6.0/resultui/html/index.html#/javascript:;)) | 0.188  （0.052-0.677） | 0.011 | 0.252  （0.053-1.203） | 0.084 | 0.695  （0.438-1.103） | 0.122 | 0.727（0.410-1.289） | 0.275 |
| Hypertension | 11.212  （3.758-33.451） | 0.001 | 9.277  （1.884-45.677） | 0.006 | 1.651（1.042-2.616） | 0.033 | 1.439（0.779-2.660） | 0.245 |
| Diabetes | 3.080  （0.722-13.133） | 0.128 | 0.044  （0.001-3.404） | 0.159 | 1.101（0.633-1.913） | 0.734 | 0.660（0.314-1.390） | 0.274 |
| Cardiovascular disease | 1.639  （0.173-15.499） | 0.666 | NA | NA | 1.682（0.973-2.905） | 0.062 | 2.088（1.027-4.246） | 0.042 |
| Chronic lung disease | NA | NA | NA | NA | 1.438（0.649-3.189） | 0.371 | 1.045（0.368-2.968） | 0.934 |
| Cerebrovascular disease | NA | NA | NA | NA | 3.601（1.477-8.780） | 0.005 | 1.753（0.539-5.701） | 0.351 |
| Chronic liver disease | 1.639  （0.173-15.499） | 0.666 | 0.841  （0.019-36.545） | 0.928 | 0.565（0.178-1.796） | 0.334 | 0.353（0.063-1.988） | 0.238 |
| Chronic kidney disease | 22.687  （2.224-231.427） | 0.008 | 28.246（0.436-182.763） | 0.116 | 2.748（0.952-7.927） | 0.062 | 2.907（0.837-10.095） | 0.093 |
| Antiviral therapy | 1.002  （0.085-11.783） | 0.687 | 1.002  （0.085-11.783） | 0.998 | 0.685（0.317-1.481） | 0.336 | 0.411（0.142-1.193） | 0.102 |
| Antibiotic therapy | 0.892  （0.143-5.572） | 0.404 | 0.892  （0.143-5.572） | 0.903 | 1.117（0.622-2.007） | 0.711 | 0.842（0.362-1.962） | 0.691 |
| Glucocorticoid therapy | 0.879  （0.141-5.469） | 0.347 | 0.879  （0.141-5.469） | 0.890 | 2.544（1.581-4.094） | ＜0.001 | 2.957（1.565-5.587） | 0.001 |
| Continuous renal replacement therapy | 14.287  （0.299-683.597） | 0.008 | 14.287  （0.299-683.597） | 0.178 | 7.370（1.538-35.324） | 0.012 | 1.388（0.120-16.011） | 0.793 |
| Oxygen inhalation | 0.704  （0.125-3.965） | 0.454 | 0.704  （0.125-3.965） | 0.691 | 0.758（0.480-1.198） | 0.236 | 0.734（0.413-1.304） | 0.292 |
| Noninvasive  mechanical ventilation | 0.944  （0.034-25.918） | 0.025 | 0.944  （0.034-25.918） | 0.973 | 4.770（2.249-10.117） | ＜0.001 | 0.739（0.191-2.855） | 0.661 |
| Invasive mechanical ventilation | 43.214（4.707-396.768） | 0.001 | NA | NA | 13.725（3.061-61.544） | 0.001 | 1.353（0.103-17.810） | 0.818 |
| Acute kidney injury | NA | NA | NA | NA | NA | NA | NA | NA |
| ARDS | 3.348  （0.917-12.225） | 0.067 | 1.577  （0.129-19.299） | 0.721 | 1.925（1.025-3.614） | 0.042 | 0.242（0.063-0.924） | 0.038 |
| Sepsis | 3.333  （0.287-38.776） | 0.336 | 0.905（0.033-24.422） | 0.952 | 34.884（8.154-149.234） | ＜0.001 | 30.877（5.509-173.048） | ＜0.001 |
| Septic shock | 22.687  （2.224-231.477） | 0.008 | 19.270（1.246-298.024） | 0.034 | 22.105（5.078-96.219） | ＜0.001 | 5.673（0.410-78.498） | 0.195 |

Abbreviations: ARDS, acute respiratory distress syndrome;  NA , not available.

^a^*P* values indicate univariate significance in [S](../../../../C:/Users/13760/AppData/Local/youdao/dict/Application/8.9.2.0/resultui/html/index.html#/javascript:;)evere patients ≤ 55 years old.

^b^*P* values indicate multivariate significance in [S](../../../../C:/Users/13760/AppData/Local/youdao/dict/Application/8.9.2.0/resultui/html/index.html#/javascript:;)evere patients ≤ 55 years old.

*^c^P* values indicate univariate significance in [S](../../../../C:/Users/13760/AppData/Local/youdao/dict/Application/8.9.2.0/resultui/html/index.html#/javascript:;)evere patients > 55 years old.

^d^*P* values indicate multivariate significance in [S](../../../../C:/Users/13760/AppData/Local/youdao/dict/Application/8.9.2.0/resultui/html/index.html#/javascript:;)evere patients > 55 years old.

*P* < 0.05 was considered statistically significant.
